# Analysis of relative bacterial activity and *lactate dehydrogenase* gene expression of caries-associated bacteria in a site-specific natural biofilm: an ex vivo study

**DOI:** 10.1007/s00784-020-03691-w

**Published:** 2020-11-23

**Authors:** Carolin Walther, Sandra Zumbülte, Christoph M. Faerber, Richard Johannes Wierichs, Hendrik Meyer-Lueckel, Georg Conrads, Karsten Henne, Marcella Esteves-Oliveira

**Affiliations:** 1 Department of Prosthetic Dentistry, Center for Dental and Oral Medicine, University Medical Center, Hamburg-Eppendorf, Martinistrasse 52, 20246 Hamburg, Germany; 2 Division of Oral Microbiology and Immunology, Department of Operative Dentistry, Periodontology, and Preventive Dentistry, RWTH Aachen University, Aachen, Germany; 3 Department of Operative Dentistry, Periodontology, and Preventive Dentistry, RWTH Aachen University, Aachen, Germany; 4 Department of Restorative, Preventive and Pediatric Dentistry, zmk bern, University of Bern, Bern, Switzerland; 5 Department of Cariology, Endodontology and Periodontology, University of Leipzig, Leipzig, Germany

**Keywords:** Dental caries/etiology, Caries diagnostic, Relative bacterial activity, 16S rRNA, *Lactobacilli*, *S. mutans*, Biofilm, *Lactate dehydrogenase gene* expression

## Abstract

**Objectives:**

Detecting bacterial activity is considered a promising approach to monitor shifts from symbiosis to dysbiosis in oral microbiome. The present study aimed at investigating both the relative bacterial activity and the lactate dehydrogenase (*ldh*) gene expression of caries-associated bacteria in a site-specific natural biofilm.

**Material and methods:**

Sixty subjects (age, mean ± SE: 30.1 ± 1.4) were allocated to two groups: caries-free subjects (CF) or caries-active subjects (CA). CF presented one sound surface (CFS, *n* = 30). CA presented two donor sites: a cavitated caries lesion (CAC, *n* = 30) and a sound reference surface (CAS, *n* = 30). Real-time quantitative PCR (q-PCR) on species or genus level and total bacteria was performed targeting the 16S gene, the 16S rRNA, the *ldh* gene, and the ldh mRNA (increasing 16S ribosomal RNA copy numbers can function as an indicator of increased energy metabolism). As the 16S rRNA abundance represents the number of ribosomes, while the 16S gene abundance represents the number of genomes, the quotient of the relative abundances functions as a measure for the relative bacterial activity (%).

**Results:**

Both *lactobacilli* and *S. mutans* showed the highest relative bacterial activity in CAC ((mean ± SE) 218 ± 60% and 61 ± 16%, respectively) and the lowest values for both sound reference surfaces (69 ± 48%; 8 ± 3%). Significant differences were found between CAC and CAS as well as between CAC and CFS for both *lactobacilli* and *S. mutans* (*p* < 0.05). The *ldh* gene expression of *lactobacilli* and *S. mutans* only showed moderate values in CAC (1.90E+03 ± 2.11E+03; 2.08E+04 ± 4.44E+04 transcripts/μl) and CFS (2.04E+03 ± 2.74E+03; 8.16E+03 ± 6.64E+03 transcripts/μl); consequently no significant differences were detected.

**Conclusion and clinical relevance:**

Caries-associated bacteria (*lactobacilli* and *S. mutans*) showed the highest relative bacterial activity in plaque of cavitated lesions, the lowest in sound surfaces, allowing the detection of a significant activity shift in health and disease for caries-active patients. However, no significant differences in *ldh* gene expression could be determined.

## Introduction

Untreated caries lesions in permanent teeth continue to be the most prevalent disease around the globe, with 2.5 billion adults being affected [1, 2]. Additionally to the already very high direct treatment costs of caries lesions, the worldwide indirect treatment costs are estimated to be around US $ 27 billion per year [2, 3]. The magnitude of these economic consequences highlights the fact that a better understanding and control of the disease can not only be extremely beneficial for public health but would also have a major global economic impact.

The disease has a multifactorial etiology, in which understanding the oral microbiome caused historically the most relevant paradigm changes [4]. According to the current accepted theory for the dental caries etiology, the oral microbiome is not an “enemy” but a natural occurrence and in symbiotic relationship with the host structures [5]. This relationship between host, environment, and microbiome is characterized by a dynamic balance, which results in oral health [6, 7]. Nevertheless, changes in the environment, like increasing the frequency of sugar consumption or a significant decrease in salivary flow, lead to increased acid production and decreased biofilm pH [8]. Persistence of this situation overtime causes a shift in the microbial community within the biofilm and selection of aciduric and acidogenic bacteria, increasing the risk of caries. In summary, there is a shift from a symbiotic relationship to dysbiosis, meaning from a healthy to a disease-prone environment [9, 10].

**Fig. 1:**
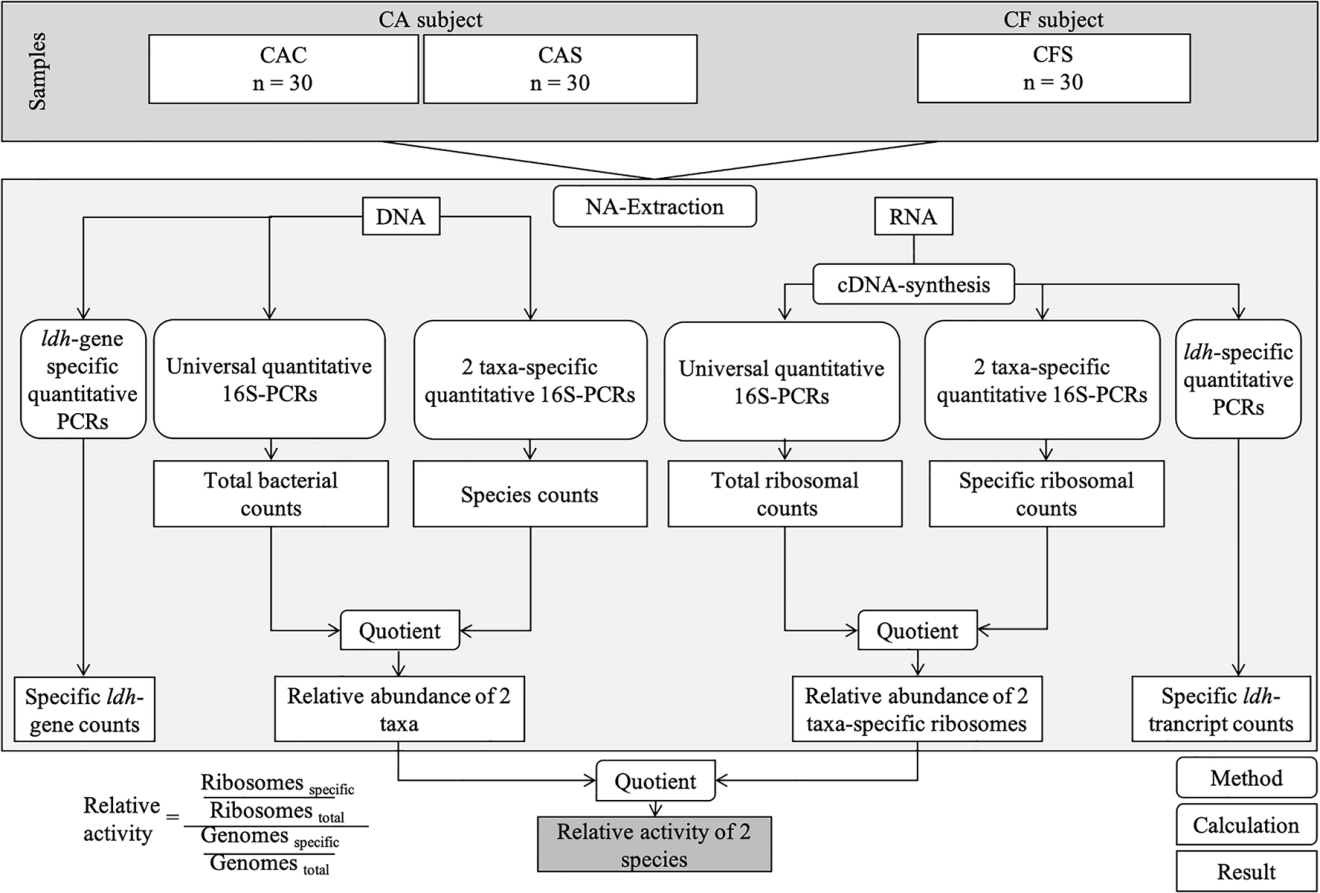
Flow diagram showing gradual progress from sampling to calculation of relative bacterial activity. Following the nucleic acid extraction, universal and specific quantitative 16S-directed PCRs were applied. The quotient of total bacterial counts and species counts represents the relative abundance of species (DNA level), whereas the quotient of total ribosome counts and specific ribosome counts represents the relative abundance of ribosomes (rRNA level). The quotient of the relative abundance of a specific ribosome and the relative abundance of the respective genome equals the relative bacterial activity

Many aspects of the taxonomic composition within the oral microbial community have been already studied [11–13]. Dental biofilm provides a wide diversity of different microbial taxa in health and disease [14]. Interestingly, the major differentiation of the bacterial taxa is within the aciduric (acid-tolerant) population selected during the caries process [15]. Besides *Streptococcus mutans* and *Lactobacillus*, recently other aciduric bacteria, like *Actinomyces* species, *Bifidobacterium*, and *Scardovia wiggsiae*, have been detected in association with caries lesions [16, 17]. Furthermore, bacterial traits related to cariogenicity (acid production and acid tolerance) are not exclusively of *Streptococcus mutans* but actually observed in a wide variety of species [9]. Thus, paying more attention to other aspects, as for example the assessment of metabolic activity/inhibition of certain virulence factors (e.g., acid production and glucan synthesis), rather than focusing on the predominant species in the oral biofilm, have become more relevant for understanding the transition between health and disease [9, 18]. However, it is still under consideration which parameters are most reliable to monitor dysbiosis within the tooth surface biofilm [15]. Most recently, technical advances in DNA and 16S ribosomal RNA (rRNA) analyses have been used to characterize oral bacteria from site- and surface-specific samples, rather than culture-based methods [19]. Nevertheless, the sheer presence of a bacterium does not automatically indicate high metabolic activity [20]. In fact, studies have demonstrated that in dental biofilm, most expressed transcripts (i.e., defined segment of DNA copied into RNA) belonged to genes for the ribosomal subunits [17, 21, 22], indicating that the functional role categories are more homogeneous across subjects, than the large interpersonal variation in species-specific transcription. Consequently, increasing 16S ribosomal RNA copy numbers can function as an indicator of increased energy metabolism. As the 16S rRNA abundance correlates with the number of ribosomes, while the 16S gene abundance is a measure for the number of genomes, the quotient of the relative abundances can function as a measure for the relative bacterial activity (Fig. 1). The interpretation is rather simple: a ratio < 100% indicates lower activity, whereas a value > 100% indicates a higher activity in comparison to the average activity in the present sample [23]. Hypothetically, the reasons for high metabolic activity among species can be either intense growth rates (anabolism) or increased substrate depletion of the cell (catabolism).

In the presence of sugar as substrate, the increased catabolism will lead to more lactic acid production induced by lactate dehydrogenase activity. Especially active caries lesions show a clear lactate-dominated acid profile, while inactive caries lesions present a variety of organic acids [24–26]. Organisms produce lactic acid from carbohydrate fermentation, and the lactate dehydrogenase (LDH) is one of the most important enzymes in the process [27, 28], catalyzing the transformation from pyruvate into lactate [29]. Consequently, we have hypothesized that the *ldh* gene could be a suitable biomarker to predict oral microbiome dysbiosis.

The methodology that we have previously established to measure the relative metabolic activity in natural and caries-associated biofilm has already shown to correlate with presence or absence of carious lesions [23], not only for some key caries-associated species, as *S. mutans*, *lactobacilli*, *Scardovia wiggsiae*, *and Bifidobacterium dentium*, but also for a non-caries-associated species, *Fusobacterium nucleatum*. Significant increased metabolic activity of caries-associated bacteria was observed in the biofilm around cavitated caries lesions, while increased metabolic activity of a non-caries-associated species was associated with the biofilm from healthy tooth surfaces. As this pattern was very clear, there are reasons to believe that further studying this method of detecting the metabolic activity in natural biofilm could support the development or be an adjuvant to others, which can monitor the oral biofilm overtime and identify patterns indicating symbiosis or dysbiosis. Thus, the investigation of its correlation with the sugar metabolism in biofilm was the next logical step.

This means that if the increased metabolic activity correlates with an increased *ldh* expression of caries-associated bacteria, it is likely to assume that the relative bacterial activity could not only detect transition from health to disease but also identify those bacteria who contribute most to the caries development. Recently, a significant correlation between the relative bacterial activities of caries-associated bacteria (*Lactobacillus paracasei*) to the *ldh* expression in co-cultures has been demonstrated [30]. However, this in vitro study worked with a “minimal version” of a planktonic microbial community. Therefore, it is still unknown, whether the distinct trends hold when analyzing a site-specific natural biofilm.

Thus, both finding a more objective method that can detect changes from a health to a disease-promoting activities in biofilm and also increasing of our understanding as regards how and when these changes occur could be of great interest for improving disease control. Therefore, the present ex vivo study aimed at investigating both the relative bacterial activity and the lactate dehydrogenase (*ldh*) gene expression of caries-associated bacteria in a site-specific natural biofilm. We formulated the following null hypothesis: There is no significant difference in the relative bacterial activity and the *ldh* expression of caries-associated bacteria in natural biofilm around cavitated caries lesions and on sound tooth surfaces.

## Material and methods

### Subjects and sampling for molecular analyses

**Fig. 2:**
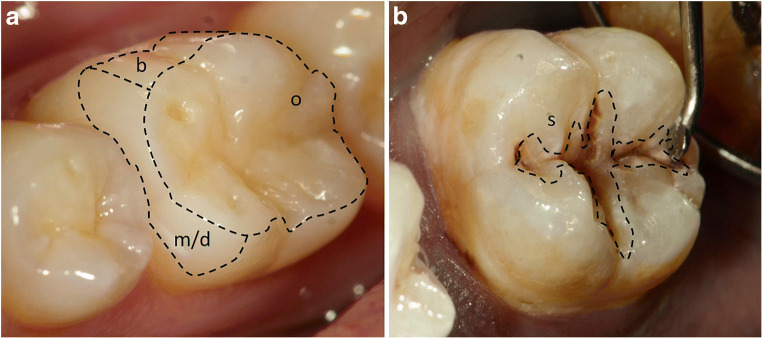
Representative picture of the sampling areas. (**a**) Sound surface: sampling from occlusal (o) and the occlusal third of the buccal (b) and the approximal (m/d) surfaces. (**b**) Cavitated caries lesion: sampling from the enamel surface at the margins of cavitation (s)

In the present study, 60 healthy adults (age, mean ± SD: 30.1 ± 1.4) were recruited from the Clinic of the Department of Operative Dentistry, Periodontology, and Preventive Dentistry, University Hospital RWTH Aachen, Germany. This study was performed in line with the principles of the Declaration of Helsinki. Approval was granted by the Ethics Committee of RWTH Aachen University (EK 017/17), and written informed consents were obtained from all participating patients. Sampling procedure and molecular biological analyses are performed according to our previous in vivo study [23]. The inclusion criteria were (1) good general and oral health (except for the caries lesions in the group of caries-active subjects) and (2) no use of antibiotics or mouthwashes within the last 3 months. Additionally, all participants were asked to refrain from food intake and oral hygiene for 12 h before plaque samples were taken. Caries-free subjects had to have a DMFT (decayed, missing, filled tooth; except for fissure sealing; absence of active non-cavitated lesions) = 0 (DMFT was determined during routine clinical examinations), whereas the caries-active subjects had to have a DMFT > 3. Subjects were allocated to two groups: CF (caries-free) or CA (caries-active) (Fig. 1). CF subjects presented one healthy sound surface (CFS, Nyvad = 0; ICDAS = 0). CA subjects presented two donor sites: a cavitated caries lesion (CAC, Nyvad = 3; ICDAS = 5/6) [31–33] and a sound reference surface (CAS), chosen in a maximal distance to the caries lesion. Two calibrated examiner (CW, CMF) collected supragingival biofilm with a sterile excavator and immediately transferred it into sterile Eppendorf tubes (1.5 ml). To obtain enough biofilm, samples from sound surfaces were taken from both the occlusal surface and the occlusal third of the buccal and approximal site. Biofilm samples from cavitated caries lesions were collected at the margin of the cavitation and not directly from the center of the lesion (Fig. 2). Subsequently all samples were weighed, frozen in liquid nitrogen, and stored at − 80 °C.

### Molecular analyses

The samples were thawed and resuspended in 200-μl bidistilled water. DNA and RNA were isolated using the DNA/RNA extraction kit NucleoSpin RNA XS (Macherey-Nagel, Düren, Germany). For the initial lysis with lysozyme and mutanolysin (3 mg lysozyme, 100 U mutanolysin, in 200 μl Tris EDTA buffer), all samples were incubated at 37 °C for 10 min, and further isolation was performed according to the manual with elution volumes of 100 μl. Subsequently, 10 μl of the RNA (1:10 dilution) was reversely transcribed into cDNA, using random hexamer primers (60 μM) with the Transcriptor First Strand cDNA Synthesis Kit (Roche, Mannheim, Germany), according to the manual. After completed reversed transcription, cDNA was stored at − 15 to − 28 °C. Specific *S. mutans* primer [23] and *Lactobacilli* primer [34] but also universal *bacteria* primer [35] were used to amplify the 16S gene and the 16S rRNA. The *ldh* gene expression was determined using self-designed L-lactate dehydrogenase gene (*ldh*) primer with a high specificity to the *ldh* gene of *S. mutans* and to the L-*ldh* variant of all oral members of the genus *Lactobacillus* (Table 1). The *ldh* gene or the respective cDNA was amplified in a qPCR with 1 μl of DNA or cDNA, the latter again diluted in bidistilled water. Primers are listed in Table 1. The q-PCRs were performed on a LightCycler 2.0 (Roche) with 1 μl of cDNA after 1:4 dilution in bidistilled water as template.

**Table 1:** Oligonucleotides used for amplification of 16S rRNA gene and *ldh* gene

Name	Sequence (5′➔3′)	Organism and fragment size (bp)	Temperature profile	Reference
Nadkarni-F	TCCTACGGGAGGCAGCAGT	All bacteria	95 °C, 10 min 95 °C, 10 s 60 °C, 10 s 72 °C, 25 s 40 cycles	Nadkarni et al. 2002
Nadkarni-R	GGACTACCAGGGTATCTAATCCTGTT			
Smut16S-81-F	CTTGCACACCGTGTTTTCT	*S. mutans* 519	95 °C, 10 min 95 °C, 10 s 55 °C, 10 s 72 °C, 25 s 50 cycles	Henne at al. 2016
Smut16S-600-R	TTTTACTCCAGACTTTCCTG			
SD-Lab-158a	GGAAACAGRTGCTAATACCG	*Lactobacilli* (genus) 549	95 °C, 10 min 95 °C, 10 s 55 °C, 10 s 72 °C, 25 s 50 cycles	Heilig et al. 2002
SD-Lab-Re	CACCGCTACACATGGAG			
Sm-ldh-1-F	TGTTCAAAATTTCAATGGCGAAG	*S. mutans* 519	95 °C, 10 min 95 °C, 10 s 60 °C, 10 s 72 °C, 25 s 40 cycles	This work
Sm-ldh-2-R	CCGATAAAGACTTCATTGAAACCA			
OralLac-ldh-1F	GGTWAACAAGAAYTTRAAGAT	*Lactobacilli* (genus) 549	95 °C, 10 min 95 °C, 30 s 60 °C, 30 s 72 °C, 30 s 40 cycles	Henne at al. 2016
OralLac-ldh-3R	AATTCWGTRTCRCCRTGTTC			
LC-ldh-1-F	GGTTAACAAGAACTTGAAGAT	*L. Casei*	95 °C, 10 min 95 °C, 10 s 60 °C, 10 s 72 °C, 20 s 40 cycles	This work
LC-ldh-2-R	CACGAACGTCTTCAAACAT			
CL-ldh-R	GTCAAYATYTCAACTGGG	*Lactobacilli* (genus) 549	95 °C, 10 min 95 °C, 10 s 60 °C, 10 s 72 °C, 20 s 40 cycles	This work
CL-ldh-F	CCWCARAARCCWGGYGAAAC			

### Relative bacterial activity

Calculation of the relative bacterial activity was described in detail previously by our group [23]. Firstly, the 16S rRNA gene copy numbers of the used taxa were obtained from the rrnDB database [36]. Subsequently the genome numbers could be determined by dividing the values of the DNA-based 16S gene q-PCR by the 16S gene copy number for the different species. In this in vivo study, for lactobacilli, we calculated a mean 16S gene copy number of 5.19 copies/genome or cell. The value for *S. mutans* was 5 copies/genome. The quotient of total bacterial genome counts and specific species genome counts represents the relative genome abundance of the respective bacterium, whereas the quotient of total ribosomal counts and specific species ribosomal counts represents the relative ribosome abundance. The quotient of the relative abundance of the respective genome and the relative abundance of a specific ribosome equals the relative bacterial activity. Values over 100% present higher ribosome content and thus higher relative activity of the regarded bacterium compared to the average relative activity of all bacterial cells; values under 100% represent a lower relative bacterial activity.

### Statistical analyses

Data were analyzed using SPSS statistical software (SPSS 25.0; SPSS, Munich, Germany). All data were not normally distributed. As CAC and CAS samples were collected within the same patient, the data is paired, and the Wilcoxon signed rank test was used to analyze significance. For the pairwise statistical analysis (CAC x CAS), the values of one subject were only included in the statistical analysis, when both data carious (CAC) and sound (CAS) from the same subject were available (Table 2). Comparisons with the CFS group did not included paired data and were therefore analyzed with the Mann-Whitney *U* test and included all available data. Finally, the correlation between the *ldh* gene expression and the relative bacterial activity was tested using the Spearman rank correlation. All tests were done at 5% significance level.

**Table 2:** Main results of molecular analyses: absolute and relative genome/ribosome counts

	Total genome	Total ribosome	*S. mutans* genome	*S. mutans* ribosome	*S. mutans ldh* gene expression	*Lactobacilli* genome	*Lactobacilli* ribosome	*Lactobacilli ldh* gene expression	*S. mutans* relative bacterial activity	*Lactobacilli* relative bacterial activity
CAC										
n*, pairwise CAC x CAS	30	30	23*	14*	4*	27*	26*	30	30	30
*n*	30	30	25	23	11	27	27	30	30	30
Mean	7.0E+05	5.2E+08	3.2E+03	1.0E+06	2.1E+04	9.6E+01	8.0E+04	1.9E+03	61.1%	218.2%
SE	1.5E+05	1.5E+08	2.7E+03	7.4E+05	1.4E+04	7.9E+01	5.1E+04	3.9E+02	16.2%	59.7%
CAS										
*n*	30	30	24	17	7	28	27	30	30	30
Mean	7.2E+05	4.3E+09	5.3E+02	4.3E+05	6.8E+05	9.2E+00	9.8E+03	2.3E+03	7.6%	69.1%
SE	1.5E+05	9.4E+08	2.5E+02	3.2E+05	7.0E+05	7.2E+00	7.9E+03	7.7E+02	3.2%	47.6%
CFS										
*n*#	30	30	18	15	4	29	28	30	30	30
Mean	3.9E+05	3.3E+08	1.6E+02	9.3E+04	8.2E+03	2.7E+00	4.8E+02	2.0E+03	27.6%	68.8%
SE	7.5E+04	9.0E+07	7.5E+01	7.7E+04	3.8E+03	3.4E-01	3.4E+02	5.1E+02	8.0%	31.5%

*As the data from CAC and CAS come from the same patient, but from different teeth (sound and carious), for the pairwise statistical analysis (CAC x CAS), the values of one subject were only included in the statistical analysis, when both data carious (CAC) and sound (CAS) from the same subject were available

#For statistical comparison between CFS x CAC and CFS x CAS, all the available data were included in the statistical analysis, as the data here were not from the same patient and so not paired

## Results

### Subjects and sampling for molecular analyses

Participants’ gender in the CA and CF groups were as follows: CA subjects (male, 26; female, 4) and CF subjects (male, 12; female, 18). Mean DMFT values were 12.5 ± 8.9 for CA subjects and 0 for CF subjects.

### Molecular analyses

**Fig. 3:**
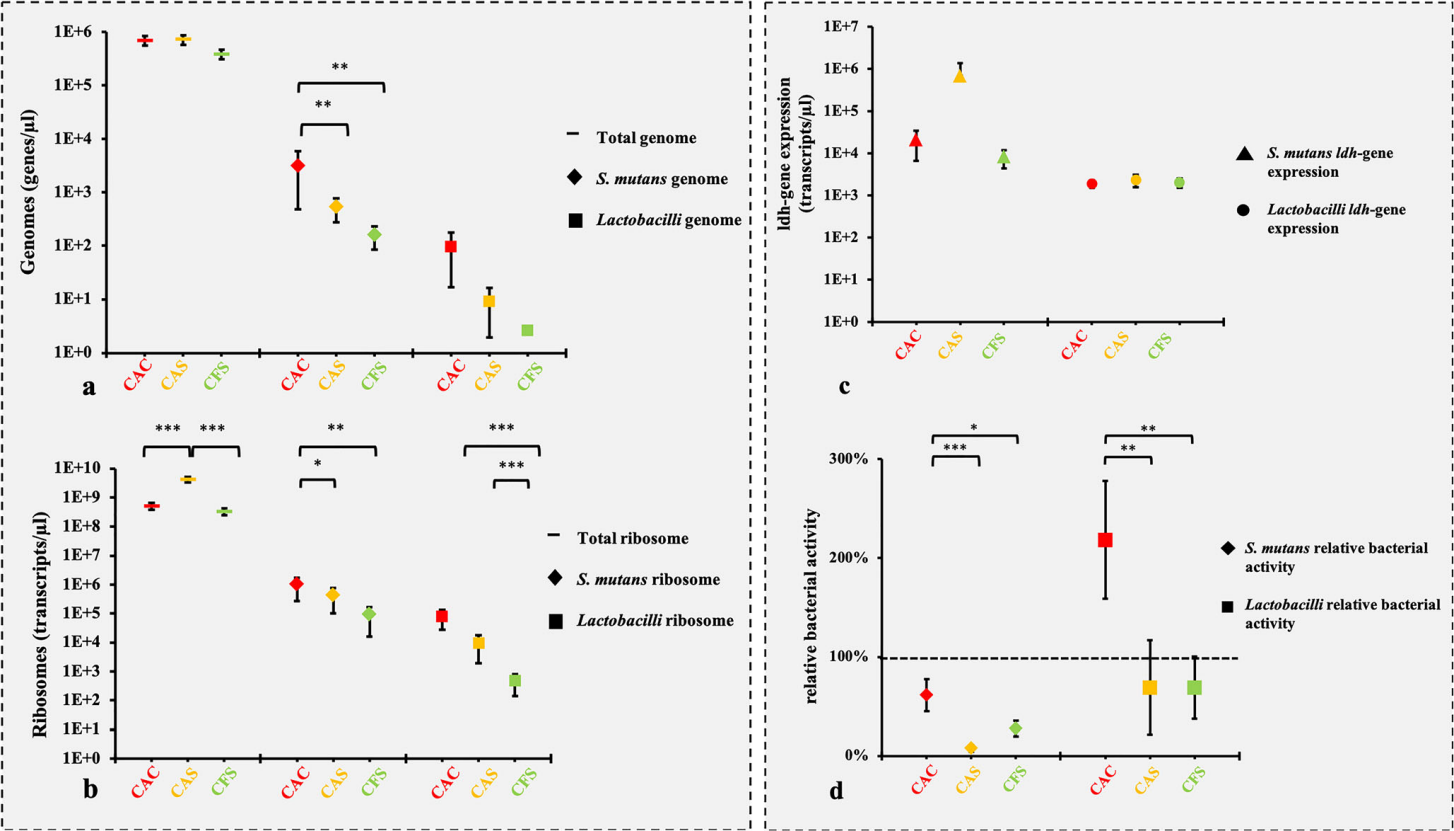
Mean numbers and standard error of genomes (**a**) and ribosomes (**b**) per microliter. Values were calculated by integrating 16S copy numbers/genome, and taxa were determined by q-PCR. CAC, CAS, and CFS present three different sampling sites. (**c**) Means of ldh gene expression for caries-associated *lactobacilli* and *S. mutans*. (**d**) Means of relative bacterial activity reveal different site-specific patterns for caries-associated *lactobacilli* and *S. mutans*. In **a** and **b,** the comparisons of the genomes and ribosomes means from CAC and CAS were done pairwise only, when data from both conditions (carious and sound) for the same subject were available. The variation between sound and carious biofilm samples within one subject (intra-subject variation) is clearly smaller than the variation between subjects (inter-subjects variation). Statistically significant differences could be detected even if the standard error bars from some groups seem to be slightly overlapping. In general overlapping error bars do not rule out statistical significance [37, 38]. *p ≤ 0.05; **p ≤ 0.01; ***p ≤ 0.001

For some subjects, either the genome, ribosome, or the *ldh* gene of *S. mutans* or *lactobacilli* could not be detected (values under the detection limit). In order to avoid overestimations, we chose the most conservative approach to analyze our measured data. This means that only the measured values were included in the statistical analyses, and no substitution for “zero” was done for the cases that no detection was possible (Table 2). For the analysis of all bacteria in dental plaque samples, mean values of total genome counts (genes/μl) differed between caries-free subjects and caries-active subjects. The highest number of bacteria counts (mean ± SE) was found for CAS (7E+5 ± 1E+5) and CAC (7E+5 ± 1E+5), and for CFS the lowest (4E+5 ± 7.5E+4) (Fig. 3a). Regarding the numbers of total ribosomal counts (transcripts/μl) the same trend was found. High means were found for CAS (4E+9 ± 9E+8), followed by CAC (5E+8 ± 1E+8) and CFS, which had the lowest means (3E+8 ± 9E+7) (Fig. 3b). Absolute and relative numbers are listed in Table 2. CAS showed though significantly higher ribosomal means than both CAC and CFS.

In the specific analysis of the caries-associated bacteria (*lactobacilli* and *S. mutans*) both, the genome and the ribosomal counts of *lactobacilli* and *S. mutans* followed the same pattern: highest counts for CAC, medium counts for CAS, and lowest counts for CFS. Differences between CAC and CAS and CAC and CFS in genome and ribosomal counts for *S. mutans* were both on a moderate significant level (*p* ≤ 0.05) (Fig. 3a/b), whereas *lactobacilli* showed highly significant differences (*p* ≤ 0.001) comparing CAC and CAS and CAC and CFS on the level of ribosomal counts. Statistically significant differences between CAC and CAS both for *S. mutans* genomes and for *S. mutans* ribosomes could be detected even if the standard error bars are slightly overlapping. In general overlapping error bars do not rule out statistical significance, as recently shown in a Nature Methods Paper [37, 38].

The *ldh* gene expression of *lactobacilli* and *S. mutans* only showed moderate values in CAC (2E+3 ± 4E+2; 2E+4 ± 1E+4 transcripts/μl), CAS (2E+3 ± 8E+2; 7E+5 ± 7E+5 transcripts/μl), and CFS (2E+3 ± 5E+2; 8E+3 ± 4E+3 transcripts/μl). Consequently, no significant differences were detected for *lactobacilli* and *S. mutans* (Fig. 3c).

### Relative bacterial activity

Both *lactobacilli* and *S. mutans* showed the highest relative bacterial activity in CAC (mean ± SE: 218 ± 60% and 61 ± 16%, respectively) and the lowest values for both sound reference surfaces (69 ± 48%; 8 ± 3%). Significant differences were found between CAC and CAS as well as between CAC and CFS for both *lactobacilli* and *S. mutans* (*p* < 0.05) (Fig. 3d).

## Discussion

The present in vivo study investigated the changes in relative bacterial activity and the lactate dehydrogenase (*ldh*) gene expression of caries-associated bacteria in a site-specific natural biofilm. In our previous in vitro study, we revealed a significant correlation between the relative bacterial activity and the *ldh* gene expression of *Lactobacillus paracasei*. Caries-associated, saccharolytic *L. paracasei* showed an increased relative bacterial activity after sucrose pulse, while non-caries-associated, weakly saccharolytic *F. nucleatum*, as a counterpart, showed a clear decrease in relative bacterial activity. Furthermore, *L. paracasei* pulsed with sucrose showed a moderate but significant positive correlation between the relative bacterial activity and the *ldh* expression [30]. Yet, it was still unclear whether the distinct trends shown in this in vitro study hold when analyzing a site-specific natural biofilm.

In order to answer this question, we designed an in vivo study model with simplified parameters: a selection of only two caries-associated taxa, *lactobacilli* and *S. mutans*. Inclusion criteria for the selected caries-associated bacteria were high acidogenic/aciduric potential, high cariogenic potential, and the ability to convert pyruvate into lactate via the lactate dehydrogenase enzyme

Additionally a study design was chosen comparing the most extreme clinical situations (clear cavitated carious lesions versus a completely sound surfaces). The reason for that is the fact that measuring the relative bacterial activity the way we presented it here and introduced it in 2016 [23] is still a quite novel approach. It is still in a proof-of-principle phase, and the main conceptual aim of this study was to gain more knowledge about the method. Meaning to test if it can detect the changes in relative bacterial activity, when they are most probably present and comparing it to the situation when they are most probably lacking (sound surfaces). Secondly, to try to correlate it more clearly with sugar metabolism, the measurement of the expression of the *ldh* gene was included. From a systematical point of view and if one wants to answer the question about the correlation of bacterial activity with health and disease, starting with the most general question and then moving step-by-step forward to the most specific ones, seems to be a structured way to approach this scientific question, as postulated in innovation funnels [39]. Analyzing ICDAS 1 and 2 lesions is for sure the long-term aim in this research line, but first a well-funded knowledge and understanding must be gained to support it. Thus, this was one of the investigations performed to achieve that.

As regards the sugar metabolism, it is known that in the final step of sugar fermentation, the enzyme lactate dehydrogenase (LDH) catalyzes the transformation from pyruvate (plus NADH) to lactate (lactic acid) plus NAD^+^. This reaction is exergonic, thermodynamically preferred, but still reversible, and lactate can be oxidized to pyruvate and NADH, if needed. Most oral acidogenic bacteria produce LDH or—on gene level—express the *ldh* gene, which directly correlates with their acid production and thereby their cariogenic property [27, 28]. We therefore hypothesized that the *ldh* gene expression could be a suitable biomarker to correlate the relative metabolic activity with the acid production within the oral microbiome in natural and site-specific dental biofilm.

In order to investigate that, we chose a simplified version of the “key players” in a natural biofilm: *lactobacilli* and *S. mutans*. However, under the conditions chosen, no significant differences in *ldh* gene expression in the biofilm of caries-active and caries-free subjects could be detected. Our null hypothesis was partially rejected (increased activity of aciduric taxa) and partially accepted (no difference in *ldh* expression).

### Relative bacterial activity

*Lactobacilli* and *S. mutans* showed the highest relative bacterial activity in CAC and lowest values for both sound reference surfaces in CAS and CFS (Fig. 3). These findings are in accordance with our previous in vivo pilot study, where caries-associated bacteria also showed the highest relative bacterial activity in caries lesions of caries-active subjects and lower activities on sound surfaces [23]. Interestingly and in accordance with the literature, in CAC samples, *lactobacilli* revealed higher overall relative bacterial activity than *S. mutans* (218 ± 60% vs. 61 ± 16%). In a mature biofilm, *Lactobacillus* is known for surviving in an extremely reduced pH environment. Eventually, *lactobacilli* can even outgrowth and exclude *S. mutans*. Thus, caries lesions are sometimes free of *S. mutans* but not or very rarely free of *lactobacilli* [40]. However, CAC presented an overall different activity profile than biofilm from CAS or CSF. The molecular biological method of the relative bacterial activity thus succeeds in generating reproducible results in this second clinical study here with noticeably more dental plaque samples from patients (*n* = 90) than in the previous pilot study.

### Molecular analyses

Both caries-associated bacteria showed high genome and ribosome counts in cavitated caries lesions, and *lactobacilli* showed lowest counts in sound surfaces in CFS. *Streptococci* inhabit different oral niches but are especially known to act as “first colonizer” of the oral cavity [41], whereas *lactobacilli* require a low pH and anaerobic retentive ecological niche [42], which can be already found in precaries lesions, but are most clearly in cavitated caries lesions. Intense catabolism of the acidogenic and aciduric bacterial groups are favored in caries lesions, and mutans streptococci and other aciduric bacteria may increase and promote lesion progression. Unexpectedly, the *ldh* gene expression of *lactobacilli* and *S. mutans* only showed moderate values in CA and CF subjects, and no significant differences could be detected. These findings are contradictory to our previous in vitro study where *L. paracasei*—after a sucrose pulse—showed a significant positive correlation with the relative bacterial activity and the *ldh* gene expression.

Comparing the primer sequences used for amplification of *ldh* genes in *S. mutans* and *lactobacilli* (Table 1) uncovers a methodological challenge. Whereas the Sm-ldh-primer could be designed relatively easy and free of any wobble position, the design of *Lactobacillus*-directed CL-ldh-primer was rather challenging. These primers were designed by comparing *ldh* paralogs of 24 *Lactobacillus* species and of related species for contrast (*Pediococcus*, *Enterococcus*, and *Streptococcus*). However, even with this effort, it was difficult to find accurate primer sequences for all variants (sometimes four in a single strain plus D-variants plus NAD-independent variants) of the *Lactobacillus ldh* gene.

Therefore, even with the best possible primer, the accurate measurement of *ldh* gene expression in *Lactobacillus* is most difficult as not encoded by a single gene. For instance, Rico et al. analyzed the genome of *L. casei* BL23 and rendered four *ldh* genes with *ldh*s 2-4 being only 49, 31, and 24% identical to *ldh*1 [43]. LDH2 (and the corresponding gene *ldh*2) had homologies to lactate/malate dehydrogenase enzymes, whereas LDH3 was most similar to L-hydroxyisocaproate dehydrogenases from many bacteria. In LDH4, sequence homology to other L-LDHs started at around amino acid 80, whereas the first N-terminal amino acids only shared a significant homology to the N-terminus of the secondary LDH from *L. lactis* [43]. Furthermore, on protein level, LDH enzymes of *lactobacilli* are regulated by phosphate, fructose 1,6-bisphosphate, pH, metal ions, and ionic strength, further complicating or hindering correlations between *ldh* gene expression, LDH synthesis, and actual lactic acid production [44]. In conclusion here, tracking of the clinically most important *lactobacilli* ldh variants would be desirable.

However, the *ldh* gene expression of *S. mutans* in CA and CF subjects did not show any increase either. This non-reactivity could point out the necessity of a short-term sucrose pulse before biofilm collection in order to activate the *ldh* gene expression. Sissons et al. already discussed the phenomena of microbiome dysbiosis in the supragingival plaque after sucrose pulse. Levels of suspected caries-associated species increased with even moderate sucrose exposure [45, 46]. Interestingly, mice fed the Western diet (high glucose, fructose, and sucrose) presented increased concentrations of multiple end products of bacterial fermentation, e.g., lactate [47]. Furthermore, recent studies proved that the sucrose pulse induced a distinct selection in the abundance pattern of sucrose degradation and glycolysis enzymes, such as L-LDH in caries-active children [48].

## Conclusion

In conclusion, under the conditions chosen, no significant differences in ldh gene expression in the biofilm of caries-active and caries-free subjects could be detected. However, caries-associated bacteria (*lactobacilli* and *S. mutans*) showed highest relative bacterial activity in the biofilm of cavitated lesions and the lowest in the biofilm of sound surfaces. Thus, indicating the potential of this technique as an adjuvant approach to monitor shifts from a healthy to a disease-prone environment in oral microbiome. Yet, molecular biology methods (e.g. primer design) are difficult and complex, and more proof of concept studies seem necessary. Future studies should consider a sucrose pulse prior sampling. Also the study design needs to consider the complete whole microbiome within the biofilm. The current strategy cannot address the issue of caries activity entirely. Thus, it is highly relevant to compare non-cavitated caries lesions with caries-free sound surfaces and to screen for caries activity in a longitudinal study design.
